# Smartphone Ownership and Eating Disorder Symptoms in Early Adolescence

**DOI:** 10.1001/jamanetworkopen.2026.37897

**Published:** 2026-09-25

**Authors:** Jason M. Nagata, Jennifer H. Wong, Jonanne Talebloo, Kyrra Engle, Kyle T. Ganson, Alexander Testa, Jinbo He, Wesley R. Barnhart, Fiona C. Baker, Jason M. Lavender

**Affiliations:** 1 Department of Pediatrics, University of California, San Francisco, San Francisco; 2 Factor-Inwentash Faculty of Social Work, University of Toronto, Toronto, Ontario, Canada; 3 Department of Management, Policy and Community Health, University of Texas Health Science Center at Houston, Houston; 4 Department of Biosciences and Bioinformatics, School of Science, Xi’an Jiaotong-Liverpool University, Suzhou, China; 5 Department of Psychology, Suffolk University, Boston, Massachusetts; 6 Center for Health Sciences, SRI International, Menlo Park, California; 7 Military Cardiovascular Outcomes Research Program, Department of Medicine, Uniformed Services University of the Health Sciences, Bethesda, Maryland; 8 The Metis Foundation, San Antonio, Texas

## Abstract

**Importance:**

Adolescents have been owning smartphones at increasingly earlier ages. The associations between age of smartphone ownership and eating disorder (ED) risk among adolescents have not been examined.

**Objective:**

To examine cross-sectional and longitudinal associations between age of smartphone ownership and ED symptoms in adolescence.

**Design, Setting, and Participants:**

The Adolescent Brain Cognitive Development (ABCD) Study used a stratified, probability-based sample of schools and recruited participants from 21 study sites across the US. Schools were oversampled based on race, ethnicity, socioeconomic status, and urbanicity to minimize systematic sampling biases in recruitment. Percentages were weighted based on American Community Survey data to represent population estimates. Cross-sectional and 2-year longitudinal analyses applied generalized linear models with a Poisson distribution and log link to ABCD Study data from years 2 to 6 (2018-2024). Data were analyzed from 2025 to 2026.

**Exposures:**

Smartphone ownership at 12 years of age (yes or no).

**Main Outcomes and Measures:**

Binge-eating symptoms, inappropriate compensatory behaviors to prevent weight gain, worry about weight gain, self-worth tied to weight, and any ED symptoms were examined separately at 12 and 14 years of age (yes or no).

**Results:**

Of 8957 participants (4712 [51.8%] male), 6225 (69.5%) owned a smartphone at 12 years of age. Of 6225 participants, the most prevalent ED symptoms were binge-eating symptoms (315 [9.2%]) at 12 years of age and compensatory behaviors (494 [20.5%]) at 14 years of age. In covariate-adjusted analyses, owning a smartphone at 12 years of age was cross-sectionally associated with a higher prevalence of binge-eating symptoms (prevalence ratio [PR], 1.61 [95% CI, 1.17-2.23]; *P* = .003), tying self-worth to weight (PR, 2.21 [95% CI, 1.03-4.74]; *P* = .04), and any ED symptom (PR, 1.36 [95% CI, 1.09-1.70]; *P* = .007). In covariate-adjusted analyses, owning a smartphone at 12 years of age was longitudinally associated with a higher risk of binge-eating symptoms (risk ratio [RR], 1.67 [95% CI, 1.11-2.52]; *P* = .01), using inappropriate compensatory behaviors to prevent weight gain (RR, 1.50 [95% CI, 1.16-1.94]; *P* = .002), tying self-worth to weight (RR, 2.03 [95% CI, 1.28-3.19]; *P* = .002), and any ED symptom (RR, 1.49 [95% CI, 1.19-1.87]; *P* < .001).

**Conclusions and Relevance:**

In this cohort study of US adolescents, smartphone ownership was associated with a variety of ED symptoms, suggesting that the timing of smartphone ownership may be associated with ED risk. Future research is needed to characterize the mechanisms underlying these observed associations.

## Key Points

**Question:**

How is age of smartphone ownership associated with eating disorder (ED) symptoms among younger adolescents?

**Findings:**

In this cohort study of 8957 adolescents, 70% of participants owned a smartphone at 12 years of age. Smartphone ownership was cross-sectionally and longitudinally associated with a greater likelihood of experiencing ED symptoms, such as binge eating and tying self-worth to weight.

**Meaning:**

Smartphone ownership at age 12 years was associated with greater eating disorder symptoms, suggesting that the timing of smartphone ownership may be associated with eating disorder risk.

## Teaser

This cohort study examines cross-sectional and longitudinal associations between smartphone ownership and eating disorder symptoms among adolescents aged 12 and 14 years in the US.

## Introduction

Millions of adolescents have eating disorders (EDs) worldwide.^1^ In the US alone, EDs are estimated to affect 3.4% to 3.8% of female-identified adolescents and 1.2% to 1.5% of male-identified adolescents.^1,2,3,4^ Notably, disordered eating that falls below the threshold of a full-syndrome ED is common. For example, a systematic review of data from 16 countries found that slightly more than 1 in 5 adolescents engage in some form of disordered eating behavior.^5^ Such symptoms can be associated with functional impairment and an increased risk of symptom progression over time. Together, these findings underscore the importance of examining ED symptoms across the full spectrum of severity in adolescent populations.^6^

Sociocultural and environmental risk factors for EDs may be especially important in adolescence, when environmental influences can have salient impacts on mental health.^7^ Smartphone use, in particular, has received extensive attention in the literature for its association with adverse outcomes, especially given that 95% of US adolescents have access to a smartphone.^8^ Among adolescents aged 8 to 16 years, smartphone ownership has been associated with problematic (compulsive) device use, psychological discomfort, and the imitation of dangerous behaviors modeled by social media influencers,^9^ with smartphone owners aged 12 years demonstrating higher rates of depression, obesity, and insufficient sleep compared with nonowners.^10^

Beyond these general health and mental health outcomes, evidence has shown that screen-based exposure is associated with disordered eating. Greater screen time, including social media, texting, and watching television, has been associated with higher levels of ED psychopathology.^11,12^ The Adolescent Brain Cognitive Development (ABCD) Study further found that each additional hour of total screen time and social media use in adolescents was associated with higher odds of compensatory behaviors to prevent weight gain, binge-eating symptoms, and self-worth tied to weight.^13^ However, despite evidence connecting both smartphone ownership and screen exposure to a range of adverse outcomes, less is known about how the age of smartphone ownership is specifically associated with the risk for ED symptoms.

Smartphone use may increase ED symptom risk through a variety of theory-informed pathways that are especially relevant during early adolescence (10-14 years of age).^14^ During early adolescence, children seek greater autonomy and peer acceptance while their social cognition and self-regulation are still developing, making them more sensitive to social evaluation and peer influence.^15,16^ It is also a period during which many, although not all, acquire their first personal smartphone.^17^

Puberty further heightens this vulnerability, as it is associated with increased body dissatisfaction and disordered eating,^18,19,20^ leaving younger adolescents especially susceptible to the appearance-focused, comparison-driven content on smartphones and social media, which could increase their risk for ED symptoms. Additionally, exposure to idealized body images through advertisements and social media content may promote appearance comparisons and ideal internalization.^21^ These processes can contribute to body dissatisfaction, a well-established risk factor for ED symptoms, consistent with the tripartite influence model.^21^

Personal smartphones may also expose adolescents to online communities that explicitly promote disordered eating. Exposure to pro-ED content (eg, “pro-ana” and “pro-mia” communities, often tagged #thinspo) has been linked to greater body dissatisfaction and disordered eating, particularly among girls,^22^ and “fitspiration” content (often tagged #fitspo) similarly has been linked to greater body dissatisfaction and appearance comparison, especially among younger users.^23,24^ Because smartphones provide private, curated access to such content, early smartphone ownership may increase exposure to these communities during a developmentally sensitive period and could be an additional pathway to ED symptom risk.^13^ Nighttime smartphone use leading to poorer sleep and disrupted homeostasis may also affect hunger and satiety regulation, as sleep dysregulation has been linked to changes in appetite signaling and emotional reactivity.^25,26^ Furthermore, viewing or engaging with content that is distressing or stressful may contribute to maladaptive coping strategies. This aligns with the affect regulation model, which suggests that individuals may use eating behaviors to manage negative emotional states that are exacerbated by both digital stressors and sleep loss.^26,27^

Notably, the developmental timing of smartphone ownership during early adolescence may be especially relevant to ED risk. In particular, having a personal smartphone may represent a distinct exposure to ED risk because it allows for more private, frequent, and individualized (eg, tailored algorithms) engagement with digital content. One study found that discrepancies between parent and adolescent reports of internalizing problems—inward-directed emotional symptoms such as anxiety, depression, and withdrawal—emerged following smartphone acquisition, with parents underestimating adolescents’ self-reported symptoms, while no comparable pattern was observed among youths without smartphones.^28^ This pattern may reflect reduced parent-child communication and fewer opportunities for parental awareness of internalizing symptoms, which are inherently less observable and rely on adolescent disclosure. Owning a personal smartphone during early adolescence may be particularly consequential, given its potential to increase unsupervised and individualized exposure to digital environments during a sensitive developmental period. Converging evidence supports associations between early digital exposure and internalizing risk; for example, among children aged 9 to 10 years, higher screen time has been associated with higher rates of internalizing disorder diagnoses, including depressive disorders, self-harm, and suicidal ideation.^29^ While major social media platforms enforce a minimum age of 13 years, this threshold can be easily circumvented, raising concerns about the feasibility of age-based protections. Given that social media use occurs primarily on smartphones, early and unsupervised access to social media has been linked to problematic screen use and prospectively associated with depressive symptoms, suicidal behaviors, and sleep disturbance in early adolescence.^30^

Although early smartphone ownership has been linked to certain adverse health outcomes such as depression, obesity, and insufficient sleep,^10^ few studies have examined whether it is associated with ED symptoms during early adolescence. As such, the purpose of our study was to examine cross-sectional and longitudinal associations between smartphone ownership and ED symptoms using data from the ABCD Study, a longitudinal, demographically diverse cohort of young adolescents in the US. We hypothesized that smartphone ownership in early adolescence would be cross-sectionally and longitudinally associated with a greater likelihood of reporting ED symptoms compared with peers who did not own a smartphone at that age.

## Methods

### Study Population

Data from year 2 (2018-2020; 10-13 years of age) to year 6 (2022-2024; 13-17 years of age) of the ongoing longitudinal ABCD Study were analyzed^31^; the periods may overlap depending on when participants were assessed in each wave. Participants missing smartphone ownership data were excluded. Centralized institutional review board approval was granted by the University of California, San Diego, and individual study sites. Written informed consent and assent were obtained from participants and parents or caregivers, respectively. We followed the Strengthening the Reporting of Observational Studies in Epidemiology (STROBE) reporting guideline for cohort studies.

### Independent Variable: Age of Smartphone Ownership

Parent- or caregiver-reported adolescent smartphone ownership data were obtained from the ABCD Screen Time Survey, with assessments conducted from year 2 (2018-2020) to year 4 (2020-2022). Parents and caregivers were asked whether their child had their own cellphone (yes or no), and if yes, reported the type of cellphone (iPhone, Android, or other; the other cellphone types were not examined in this study) and the age at which the child first obtained the device.^28^ Because our primary exposure focused on smartphone ownership, analyses were restricted to participants who owned or did not own a smartphone. In the present study, we focused on smartphone ownership at 12 years of age, defined as 12.00 to 12.99 years.^32^

### Dependent Variable: ED Symptoms

The Kiddie Schedule for Affective Disorders and Schizophrenia was used to assess adolescent-reported ED symptoms at years 2 (2018-2020), 4 (2020-2022), and 6 (2022-2024).^33^ Participants reported whether they experienced the following ED symptoms (yes or no): (1) binge eating, (2) inappropriate compensatory behaviors to prevent weight gain, (3) worry about weight gain, and (4) self-worth tied to weight. We focused on ED symptoms at 12 and 14 years of age.

### Potential Confounders

Covariates assessed at year 2 were included in the analysis to account for potential confounding associated with smartphone ownership and ED symptoms.^34^ These covariates included parent- or caregiver-reported sex (female [reference] or male), race (Black, White [reference], or other [including American Indian or Alaska Native, Asian, Native Hawaiian or Other Pacific Islander, multiracial, not reported, or other]), ethnicity (Hispanic or non-Hispanic [reference]), annual household income (≤$49 999, $50 000-$99 999, or ≥$100 000 [reference]), highest parent- or caregiver-attained educational level (high school or less, or college or more [reference]), and study site. Race and ethnicity data were included to characterize the study population and provide context for the demographic composition of the sample. Parent- or caregiver-reported attention-deficit/hyperactivity and depressive symptoms from the Child Behavior Checklist, adolescent-reported pubertal status and parental monitoring, and body weight were included.^35,36,37^ Last, to isolate the influence of smartphone ownership, we adjusted for the parent- or caregiver-reported ownership of tablets, iPods or similar devices, laptops, and smartwatches, in line with prior approaches.^10^ Due to research identifying sex-based differences in smartphone ownership and ED symptoms, we tested for an interaction between sex and smartphone ownership.^17^

### Statistical Analysis

Data were analyzed from 2025 to 2026. Analyses were conducted in Stata, version 18.0 (StataCorp LLC). We conducted 2 sets of statistical analyses using multiple covariate-adjusted generalized linear models with a Poisson distribution and log link. We examined (1) smartphone ownership and ED symptoms at 12 years of age, following prior methods (cross-sectional analysis),^10^ and (2) smartphone ownership at 12 years of age and ED symptoms at 14 years of age, controlling for ED symptoms at 12 years of age, to evaluate the temporal nature of these associations (longitudinal analysis). We tested for interaction with smartphone ownership by sex. Sampling weights were applied to analyses to approximate the sociodemographic characteristics reported in the American Community Survey by the US Census Bureau.^38^ All tests were 2 sided with statistical significance defined as *P* < .05.

## Results

A total of 8957 adolescents were included in the analysis (4245 [48.2%] female and 4712 [51.8%] male; 1364 [14.2%] Black, 6058 [70.4%] White, and 1534 [15.3%] other race; 1792 [23.6%] Hispanic). Of these, 6225 adolescents (69.5%) owned a smartphone at 12 years of age, including 4198 (64.5%) who owned an iPhone and 2027 (35.5%) who owned an Android; 3122 (51.2%) were female and 3103 (48.8%) were male; 1095 (16.2%) were Black, 4025 (67.9%) were White, 1105 (15.8%) were of other race; and 1348 (25.6%) were Hispanic (Table 1). Compared with adolescents who did not own a smartphone at 12 years of age, those who owned a smartphone were more likely to be female, Black, and Hispanic; to have lower household income and lower parental educational attainment; to be in a more advanced pubertal stage; and to own other personal devices (eg, laptops and smartwatches). Nonowners were more likely to own a tablet or an iPod. Among the 8957 adolescents, the most and least prevalent ED symptoms at 12 years of age were binge eating (405 [8.2%]) and worry about weight gain (73 [1.8%]), respectively. Among the 6225 adolescents who owned a smartphone, the most prevalent ED symptoms were binge eating at 12 years of age (315 [9.2%]) and compensatory behaviors at 14 years of age (494 [20.5%]); nearly one-quarter (619 [24.2%]) reported at least 1 ED symptom. Smartphone owners had a higher prevalence of all ED symptoms than nonowners at 12 and 14 years of age. Compared with included participants, excluded participants were more likely to identify as a member of a racial minority group and to have parents with lower annual incomes and lower educational levels (eTable 2 in Supplement 1). We stratified results by sex for outcomes where there was evidence of significant effect modification by sex on the associations between smartphone ownership and ED symptoms. In adjusted models with cross-sectional analysis (eTable 3 in Supplement 1), female adolescents who owned a smartphone reported greater worry about weight gain than males.

**Table 1.** Participant Characteristics

Characteristic	Study group, No. (weighted %)^a^			*P* value
	Overall (N = 8957)	No smartphone ownership (n = 2732)	Smartphone ownership (n = 6225)	
Smartphone type				
iPhone	NA	NA	4198 (64.5)	NA
Android	NA	NA	2027 (35.5)	
Sex				
Female	4245 (48.2)	1123 (41.3)	3122 (51.2)	<.001
Male	4712 (51.8)	1609 (58.7)	3103 (48.8)	
Race				
Black	1364 (14.2)	269 (9.4)	1095 (16.2)	<.001
White	6058 (70.4)	2033 (76.4)	4025 (67.9)	
Other^b^	1534 (15.3)	429 (14.2)	1105 (15.8)	
Ethnicity				
Hispanic	1792 (23.6)	444 (18.9)	1348 (25.6)	.001
Non-Hispanic	7147 (76.4)	2281 (81.1)	4866 (74.4)	
Household income (year 2)				
≤$49 999	2022 (31.4)	489 (26.5)	1533 (33.4)	.001
$50 000-$99 999	2222 (30.5)	690 (30.8)	1532 (30.4)	
≥$100 000	4023 (38.1)	1362 (42.7)	2661 (36.2)	
Parent or caregiver’s highest educational attainment (year 2)				
High school education or less	1164 (15.6)	272 (12.7)	892 (16.8)	.03
College education or more	7781 (84.4)	2458 (87.3)	5323 (83.2)	
Child Behavior Checklist t score (year 2), mean (SD)^c^				
Attention-deficit/hyperactivity symptoms	53.4 (5.5)	53.6 (5.8)	53.2 (5.4)	.12
Depressive symptoms	54.1 (6.1)	54.1 (6.1)	54.1 (6.1)	.68
Device ownership (year 2)				
Tablet	3960 (44.4)	1233 (46.1)	2727 (43.7)	.21
Laptop	3332 (36.9)	719 (26.5)	2613 (41.4)	<.001
iPod	980 (10.5)	443 (16.0)	537 (8.2)	<.001
Smartwatch	502 (5.4)	75 (2.5)	427 (6.7)	<.001
Pubertal status (year 2)				
Prepubertal or early pubertal	3184 (37.7)	1189 (46.1)	1995 (34.2)	<.001
Midpubertal, late pubertal, or post pubertal	4900 (62.3)	1304 (53.9)	3596 (65.8)	
Body weight, mean (SD), kg	49.5 (14.8)	46.7 (12.7)	50.8 (15.5)	<.001
Parental monitoring score (year 2), mean (SD)^d^	4.5 (0.5)	4.4 (0.5)	4.5 (0.5)	.25
ED symptoms (12 years of age)				
Binge eating	405 (8.2)	90 (6.0)	315 (9.2)	.003
Inappropriate compensatory behaviors to prevent weight gain	342 (7.3)	74 (5.3)	268 (8.2)	.005
Worry about weight gain	73 (1.8)	16 (1.2)	57 (2.0)	.02
Self-worth tied to weight	91 (2.2)	15 (1.1)	76 (2.6)	.001
Any ED symptom	706 (14.7)	164 (11.3)	542 (16.1)	.002
ED symptoms (14 years of age)				
Binge eating	285 (8.3)	67 (6.5)	218 (9.2)	<.001
Inappropriate compensatory behaviors to prevent weight gain	632 (18.2)	138 (12.7)	494 (20.5)	<.001
Worry about weight gain	76 (2.1)	20 (1.6)	56 (2.3)	.36
Self-worth tied to weight	229 (6.5)	44 (3.5)	185 (7.8)	<.001
Any ED symptom	799 (21.6)	180 (15.5)	619 (24.2)	<.001

Abbreviations: ED, eating disorder; NA, not applicable.

^a^ Sampling weights based on the American Community Survey were used to represent population estimates. Counts are unweighted and percentages are weighted, so raw counts may not match percentages. Category totals may not sum to the sample size within each column because of missing data.

^b^ Includes American Indian or Alaska Native, Asian, Native Hawaiian or Other Pacific Islander, multiracial, not reported, or other.

^c^ Scores ranged from 50 to 90, with higher scores indicating greater attention-deficit/hyperactivity and depressive symptoms.

^d^ Responses were rated on a 5-point Likert scale from 1 (never) to 5 (always or almost always).

Owning a smartphone at 12 years of age was associated with a higher prevalence of reporting binge-eating symptoms (prevalence ratio [PR], 1.61 [95% CI, 1.17-2.23]; *P* = .003), tying self-worth to weight (PR, 2.21 [95% CI, 1.03-4.74]; *P* = .04), and any ED symptom (PR, 1.36 [95% CI, 1.09-1.70]; *P* = .007) in cross-sectional analysis (Table 2). In longitudinal analysis, owning a smartphone at 12 years of age was associated with a higher risk of reporting binge-eating symptoms (risk ratio [RR], 1.67 [95% CI, 1.11-2.52]; *P* = .01), using inappropriate compensatory behaviors to prevent weight gain (RR, 1.50 [95% CI, 1.16-1.94]; *P* = .002), tying self-worth to weight (RR, 2.03 [95% CI, 1.28-3.19]; *P* = .002), and any ED symptom (RR, 1.49 [95% CI, 1.19-1.87]; *P* < .001) at 14 years of age.

**Table 2.** Associations Between Smartphone Ownership and ED Symptoms in the Adolescent Brain Cognitive Development Study^a^

ED symptom	Cross-sectional analysis^b^		Longitudinal analysis^c^	
	PR (95% CI)	*P* value	RR (95% CI)	*P* value
Binge eating	1.61 (1.17-2.23)	.003	1.67 (1.11-2.52)	.01
Inappropriate compensatory behaviors	1.31 (0.94-1.83)	.11	1.50 (1.16-1.94)	.002
Worry about weight gain	1.30 (0.57-2.95)	.53	1.02 (0.51-2.05)	.95
Self-worth tied to weight	2.21 (1.03-4.74)	.04	2.03 (1.28-3.19)	.002
Any	1.36 (1.09-1.70)	.007	1.49 (1.19-1.87)	<.001

Abbreviations: ED, eating disorder; RR, risk ratio; PR, prevalence ratio.

^a^ Sampling weights were applied to yield estimates based on the American Community Survey from the US Census. Models represent the abbreviated output from the generalized linear models with adjustment for sex, race, ethnicity, year 2 household income, highest parental educational attainment, attention-deficit/hyperactivity symptoms, depressive symptoms, tablet ownership, laptop ownership, iPod ownership, smartwatch ownership, parental monitoring, pubertal status, body weight, and study site.

^b^ Analyzes smartphone ownership and ED symptoms at 12 years of age.

^c^ Analyzes smartphone ownership at 12 years of age and ED symptoms at 14 years of age. Additionally adjusted for ED symptoms at 12 years of age.

## Discussion

In this demographically diverse cohort of early adolescents, we found that, consistent with our hypothesis, smartphone ownership at 12 years of age was cross-sectionally associated with a higher likelihood of reporting binge-eating symptoms, tying self-worth to weight, and any ED symptom. Smartphone ownership at 12 years of age was also longitudinally associated with a higher likelihood of reporting binge-eating symptoms, using inappropriate compensatory behaviors to prevent weight gain, tying self-worth to weight, and any ED symptom at 14 years of age. In analyses stratified by sex, the cross-sectional association between smartphone ownership at 12 years of age and worry about weight gain demonstrated a significant increase among females but not males, suggesting that early-adolescent females may be particularly susceptible to weight-related concerns associated with smartphone ownership. Our findings are notable given that early adolescence, when self-regulation is still maturing and sensitivity to social evaluation is heightened, coincides with puberty, which is a time of increased ED risk.^7,18^ These findings build on evidence that smartphone ownership is commonly established during early adolescence; that early smartphone ownership and problematic screen use are linked to adverse mental health, sleep, and behavioral outcomes in youths^10,17,30^; and that disordered eating itself is prevalent in adolescents and prospectively tied to screen-based exposures.^5,12,39^ Together, these findings suggest that smartphone ownership may be an additional modifiable correlate of ED risk during this sensitive period.^39^ Compared with other studies that have largely focused on screen use metrics or problematic smartphone use, our study focused on smartphone ownership and ED symptoms among adolescents. Worry about weight gain was not associated with smartphone ownership in adjusted models. Weight gain concerns were not highly prevalent in this sample, which may have limited our ability to detect differences by smartphone ownership.

The observed associations may be explained by several potential mechanisms. Unsupervised personal smartphones offer adolescents more opportunities for uninterrupted, autonomous access to digital content^40^ and may increase exposure to social comparison and problematic social media use, which has been found to be associated with body dissatisfaction and disordered eating.^13,41^ Similar reward-related and impulsive processes have also been implicated in binge eating, which has been conceptualized as sharing features with addiction and problematic screen use.^13^ Thus, the association between early smartphone ownership and ED symptoms may be explained by increased exposure to appearance-focused content and social comparison, as well as reduced parental regulation during this developmental period.

These influences may also accumulate over time and be shaped by developmental timing. For instance, the longer adolescents are exposed to idealized body content on social media, the greater the associated risk of body dissatisfaction and disordered eating, consistent with the tripartite influence model.^21,42,43^ Appearance-related pressures are reflected in emerging online trends such as “looksmaxxing,” which has surged in popularity and media coverage, promotes intense optimization of physical appearance, and has been linked to dangerous practices and physical and mental harms.^44^ Last, nighttime smartphone use may disrupt sleep and homeostasis, which has been linked to alterations in appetite regulation and emotional reactivity that may increase vulnerability to ED symptoms.^25,26^

EDs are serious, potentially life-threatening conditions that are nonetheless often preventable and responsive to early interventions.^45^ The high symptom burden observed among smartphone owners in this study—24.2% of adolescents aged 14 years reporting at least 1 ED symptom—suggests the urgency of prevention efforts. Clinicians may consider screening for smartphone ownership and social media use when assessing ED and mental health risks.

Individual-level considerations also connect to a broader policy debate over youth access to smartphones and social media. Professional bodies, including the American Academy of Pediatrics, encourage families to delay and individualize smartphone ownership and to use tools such as the Family Media Plan.^46,47^ Governments have also taken action, such as Australia’s law that set a minimum age of 16 years for social media accounts, effective December 2025.^48^ Our finding that smartphone ownership at 12 years of age was associated with elevated risk of ED symptoms is consistent with efforts to delay early, independent access of smartphones. Future studies are needed to better characterize the mechanisms underlying the association between early smartphone ownership and subsequent ED symptoms in adolescents, including potential roles of social media exposure, sleep disruption, and body image–related processes. Future research with larger samples should examine whether transitions in smartphone ownership are associated with changes in ED symptoms over time, particularly among adolescents who newly acquire smartphones.

Several strengths of this study should be noted, including the analysis of a large, diverse longitudinal cohort of US adolescents and the examination of ED symptoms, which allows for the detection of disordered eating that may not meet full diagnostic criteria. Additionally, we found longitudinal associations between the age of smartphone ownership and ED symptoms, an area that has not been previously explored in the literature. We also used the validated Kiddie Schedule for Affective Disorders and Schizophrenia to assess ED symptoms, which is a particular strength given the limited availability of validated measures for subclinical ED symptoms in population-based samples of youths.

### Limitations

This study has several limitations. The study used adolescent-reported ED symptoms, which may be subject to reporting bias. Despite adjusting for numerous potential confounders, residual confounding may remain. Eating disorders may precipitate from other psychosocial factors, including school or online bullying, neurodevelopmental conditions, and pre-existing or emerging mental health problems, which were not accounted for in this analysis. Although the longitudinal study design was a strength, smartphone ownership was not randomized and reverse causation cannot be ruled out, so causality cannot be established. In addition, although we accounted for ownership of other devices (ie, tablets, iPods or similar devices, laptops, and smartwatches), smartphone ownership did not capture the content consumed by adolescents or patterns of use, which may be relevant in determining the development of ED symptoms. Children with earlier smartphone ownership may differ systematically from those without smartphones in unmeasured characteristics associated with ED symptoms, potentially contributing to observed associations. Participants excluded due to missing data may have also differed systematically from the analytic sample, for example, being a member of a racial or ethnic minority group and having parents with lower annual incomes or lower educational levels, potentially introducing sampling bias and limiting the generalizability of our findings to these populations. Last, ED symptoms were coded dichotomously, limiting the ability to capture variation in symptom severity and other aspects of ED pathology.

## Conclusions

In this cohort study of adolescents, the findings highlight the need to understand the potential downstream influences of the age of smartphone ownership among adolescents, particularly given that smartphone ownership is increasingly occurring at younger ages. Parents and caregivers may benefit from guidance around adolescent smartphone ownership, including consideration of age-appropriate limits and supervision of early device access. At the policy level, these findings underscore the importance of developing evidence-informed recommendations regarding smartphone ownership in early adolescence. Collaborative efforts among families, schools, clinicians, public health programs, and policymakers may help support healthy technology use and reduce potential risks associated with early smartphone access.

## Notes

Supplement 1. **eMethods.** Study Design, Measures, and Statistical Analysis **eTable 1.** KSADS-5 Assessment of Eating Disorder Symptoms in the Adolescent Brain Cognitive Development (ABCD) Study **eTable 2.** Comparison of Participants Included vs Excluded in the Adolescent Brain Cognitive Development (ABCD) Study (N = 11 962) **eTable 3.** Cross-sectional Smartphone Ownership Associations With Eating Disorder Symptoms Stratified by Sex in the Adolescent Brain Cognitive Development (ABCD) Study **eTable 4.** Distribution of Age of Smartphone Ownership by Study Year in the Adolescent Brain Cognitive Development (ABCD) Study **eTable 5.** Cross-sectional Associations Between Smartphone Ownership at Age 12 and Eating Disorder Symptoms at Age 12 in the Adolescent Brain Cognitive Development (ABCD) Study **eTable 6.** Longitudinal Associations Between Smartphone Ownership at Age 12 and Eating Disorder Symptoms at Age 14 in the Adolescent Brain Cognitive Development (ABCD) Study **eTable 7.** Cross-sectional Associations Between Smartphone Ownership at Age 12 and Eating Disorder Symptoms at Age 12 in the Adolescent Brain Cognitive Development (ABCD) Study **eTable 8.** Longitudinal Associations Between Smartphone Ownership at Age 12 and Eating Disorder Symptoms at Age 14 in the Adolescent Brain Cognitive Development (ABCD) Study
eReferences

Supplement 2. Data Sharing Statement
