# Supplementary material for: Smartphone Ownership and Eating Disorder Symptoms in Early Adolescence
Source: JAMA Netw Open. 2026 Sep 25;9(9):e2637897. doi: 10.1001/jamanetworkopen.2026.37897 (PMC13615465; doi:10.1001/jamanetworkopen.2026.37897)
Supplement: Supplementary file 1 [file jamanetwopen-e2637897-s001.pdf]

Supplementary Online Content

Nagata JM, Wong JH, Talebloo J, et al. Smartphone ownership and eating disorder symptoms in early adolescence. *JAMA Netw Open.* 2026;9(9):e2637897. doi:10.1001/jamanetworkopen.2026.37897

eMethods. Study Design, Measures, and Statistical Analysis

eTable 1. KSADS-5 Assessment of Eating Disorder Symptoms in the Adolescent Brain Cognitive Development (ABCD) Study

eTable 2. Comparison of Participants Included vs Excluded in the Adolescent Brain Cognitive Development (ABCD) Study (N = 11 962)

eTable 3. Cross-sectional Smartphone Ownership Associations With Eating Disorder Symptoms Stratified by Sex in the Adolescent Brain Cognitive Development (ABCD) Study

eTable 4. Distribution of Age of Smartphone Ownership by Study Year in the Adolescent Brain Cognitive Development (ABCD) Study

eTable 5. Cross-sectional Associations Between Smartphone Ownership at Age 12 and Eating Disorder Symptoms at Age 12 in the Adolescent Brain Cognitive Development (ABCD) Study

eTable 6. Longitudinal Associations Between Smartphone Ownership at Age 12 and Eating Disorder Symptoms at Age 14 in the Adolescent Brain Cognitive Development (ABCD) Study

eTable 7. Cross-sectional Associations Between Smartphone Ownership at Age 12 and Eating Disorder Symptoms at Age 12 in the Adolescent Brain Cognitive Development (ABCD) Study

eTable 8. Longitudinal Associations Between Smartphone Ownership at Age 12 and Eating Disorder Symptoms at Age 14 in the Adolescent Brain Cognitive Development (ABCD) Study

eReferences

This supplementary material has been provided by the authors to give readers additional information about their work.

© 2026 Nagata JM et al. *JAMA Network Open*.

eMethods. Study Design, Measures, and Statistical Analysis

Study Design

The study follows 11,962 early adolescents recruited from 21 study sites in the U.S. to examine the overall health, cognitive development, and behavioral patterns in adolescents.[1] The Adolescent Brain Cognitive Development (ABCD) Study utilized a stratified, probability sample of schools across the U.S. to capture demographic diversity and obtain a representative U.S. sample.[1] Participants were recruited through public and private schools while considering factors such as sex, race, ethnicity, socioeconomic status, and urbanicity, to prevent sampling bias. Although the ABCD Study was not designed to produce nationally representative estimates, its sampling strategy was developed to approximate the demographic diversity of the U.S. adolescent population. Additional details on the ABCD Study measures, recruitment procedures, and sample characteristics have been reported elsewhere.[1]

The current study used data from the ABCD 6.0 release. The 6.0 release was made available in June 2025 and refers to the sixth publicly available release of data from the ABCD Study.[2] This release provides updated cumulative visit data and includes new study years up to Year 5 (2021–2023).[2] Participants missing smartphone ownership data (n=3,005) were excluded, resulting in a sample of 8,957 adolescents. The last observation carried forward method was used to impute remaining missing values using the most recent observation for household income, parent education, study site, attention-deficit/hyperactivity symptoms, and depressive symptoms. Centralized institutional review board approval was granted by the University of California, San Diego, and individual study sites. Written informed consent and assent were respectively obtained by parents/caregivers and participants. This study followed the Strengthening the Reporting of Observational Studies in Epidemiology (STROBE) reporting guidelines.

Measures

Independent variable: Age of smartphone ownership

Parents were asked whether their child had their own cellphone (yes/no), and if so, reported the type of cellphone (iPhone, Android, other). Other cellphone types were not examined in this study.[3]

Smartphone ownership at age 12, defined as 12.00–12.99 years, was determined based on the first available wave in which the child was 12 years old, with priority given to Year 2, followed by Year 3, and Year 4 if earlier data were not available. Age 12 was selected because it marks the developmental period during which many children first gain independent access to smartphones,[4] and because most participants were 12 years old at Year 2, ensuring adequate sample sizes and statistical power for analyses.

Dependent variable: Eating disorder (ED) symptoms

Adolescent-reported ED symptoms were assessed using the computerized Kiddie Schedule for Affective Disorders and Schizophrenia (KSADS-5) interview, a validated computerized tool for diagnostically categorizing child and adolescent mental health issues according to Diagnostic and Statistical Manual-5 criteria.[5]

The exact survey questions, operationalization, and coding of each symptom are presented in eTable 1. Each ED symptom was assessed and coded as a single dichotomous (yes/no) variable in the ABCD dataset. An "any ED symptom" variable was created to reflect the presence of at least one of the four ED symptoms. In the current study, we focused on ED

© 2026 Nagata JM et al. *JAMA Network Open*.

symptoms at age 12 (defined as 12.00–12.99 years), and age 14 (defined as 14.00–14.99 years). ED symptoms were determined based on the first available wave in which the child was 12 years old and 14 years old.

Potential Confounders

Covariates were assessed at Year 2, when the mean age of participants was approximately 12, to align covariate measurement with the exposure of smartphone ownership at age 12. Race and ethnicity were reported by a parent or caregiver/guardian using demographic questionnaires administered as part of the ABCD Study. Race was collapsed from the original National Institute of Health 7-level classification (American Indian/Alaska Native, Asian, Black [African American], More than One Race, Native Hawaiian or Other Pacific Islander, Other [Unknown or not reported], and White) into three categories (White, Black, and other race) due to sparse cell sizes, which limited the stability of estimates for individual categories. Other race includes American Indian or Alaska Native, Asian, more than one race, Native Hawaiian or Other Pacific Islander, race not reported, and other (self-described). Parent/caregiver-reported sociodemographics, including, sex, race, ethnicity, annual household income, highest parent/caregiver education, and study site, were adjusted for since these covariates have been associated with smartphone ownership and ED symptoms in prior research.[3,6]

Parent/caregiver-reported attention-deficit/hyperactivity and depressive symptoms from the Child Behavior Checklist were included as covariates due to previous associations with smartphone ownership and binge-eating disorder.[7,8]

Pubertal status, measured on a 1–5 Likert-type scale from pre-pubertal to post-pubertal, was included due to associations with screen time and disordered eating.[9,10] In this analysis, we collapsed the responses into two categories due to sparse cells: pre/early-pubertal and mid/late/post-pubertal.

Body weight (pounds) was included as a covariate due to known associations with body mass index and binge-eating disorders in adolescents.[11] Body weight was measured three times and then averaged.

Adolescent-reported parental monitoring, assessed as the mean score of five items from the Parental Monitoring scale, was controlled for. Parental monitoring example items include, “How often do your parents/guardians know where you are?” and “How often do your parents know who you are with when you are not at school and away from home?” Parental monitoring was controlled for due to previously reported associations with mobile phone use and EDs.[12,13]

Statistical Analysis

eTable 3 shows the cross-sectional smartphone ownership associations with ED symptoms stratified by sex. The distribution of age of smartphone ownership by study year is shown in eTable 4. The unadjusted model results for the cross-sectional analyses are shown in eTable 5 and the longitudinal analyses are shown in eTable 6. The full adjusted model results for the cross-sectional analyses shown in eTable 7 and the longitudinal analyses are shown in eTable 8.

© 2026 Nagata JM et al. *JAMA Network Open*.

eTable 1. KSADS-5 Assessment of Eating Disorder Symptoms in the Adolescent Brain Cognitive Development (ABCD) Study

Symptom	Question	Operationalization and Coding
Binge Eating	In the past two weeks, how often have you had eating binges, when you lost control of your eating and ate way more than you needed, because you were unable to stop yourself from eating?	Participants selected responses as follows: Not at all, rarely, several days, more than half the days, and nearly every day. Participants who reported any binge eating were coded as having binge eating symptoms.
Worry About Weight Gain	In the past two weeks, how often have you been preoccupied with your weight or worrying a lot about being fat?	Participants selected responses as follows: Not at all, rarely, several days, more than half the days, and nearly every day. Those who responded "more than half the days" or "nearly every day" were coded as worrying about weight gain.
Self-Worth Tied to Weight	Do you feel like your self-worth is tied to your weight?	Participants who responded "yes" were coded as feeling self-worth tied to weight.
Compensatory Behaviors to Prevent Weight Gain	Please note below all the different methods that you have used to control your weight.	Participants were asked to select from the following behaviors: diet pills, laxatives, water pills, throwing up, exercising a lot, only eating foods or drinks with minimal calories (e.g., carrots, celery, zero calorie drinks), or other (fill in). Those who selected at least one response option were coded as engaging in inappropriate behaviors to prevent weight gain.

© 2026 Nagata JM et al. *JAMA Network Open*.

eTable 2. Comparison of Participants Included vs Excluded in the Adolescent Brain Cognitive Development (ABCD) Study in Year 2 (2018–2020) (N=11,962)

Sociodemographic characteristics	Included (n=8,957)	Excluded (n=3,005)	p
Sex			
Female	4,245 (48.2%)	1,433 (50.4%)	0.09
Male	4,712 (51.8%)	1,478 (49.6%)	
Race			
Black	1,364 (14.2%)	565 (17.6%)	<0.001
Other	1,534 (15.3%)	598 (19.7%)	
White	6,058 (70.4%)	1,747 (62.7%)	
Ethnicity			
Hispanic	1,792 (23.6%)	653 (26.1%)	0.20
Non-Hispanic	7,147 (76.4%)	2,232 (73.9%)	
Household income			
$49,999 or less	2,022 (31.4%)	848 (40.8%)	<0.001
$50,000 to $99,999	2,222 (30.5%)	665 (29.3%)	
$100,000 or greater	4,023 (38.1%)	1,041 (29.9%)	
Parent/caregiver's highest education			
High school education or less	1,164 (15.6%)	578 (22.7%)	<0.001
College education or more	7,781 (84.4%)	2,327 (77.3%)	

Sampling weights based on the American Community Survey were used to represent population estimates. Counts are unweighted and percentages are weighted, so raw counts may not match percentages.

© 2026 Nagata JM et al. *JAMA Network Open*.

eTable 3. Cross-sectional Smartphone Ownership Associations With Eating Disorder Symptoms Stratified by Sex in the Adolescent Brain Cognitive Development (ABCD) Study[a]

	Worry about weight gain at age 12				
	Male (N=1,781)			Female (N=1,562; reference)	
	PR (95% CI)	p	p[b]	PR (95% CI)	p
Smartphone ownership at age 12	0.64 (0.17, 2.37)	0.504	**0.04**	**2.96 (1.15, 7.61)**	**0.03**

[a]Bold indicates p<0.05. PR=prevalence ratio from generalized linear models. Models represent the abbreviated output from the generalized linear models including adjustment for age, sex, race and ethnicity, household income, parent education, and study site in addition to all variables listed. Sample weights from the Adolescent Brain Cognitive Development Study were applied based on the American Community Survey from the US Census.

[b]P-value for the smartphone ownership*sex interaction term coefficient

© 2026 Nagata JM et al. *JAMA Network Open*.

eTable 4. Distribution of Age of Smartphone Ownership by Study Year in the Adolescent Brain Cognitive Development (ABCD) Study

	Year 2 (N=6,738)	Year 3 (N=7,677)	Year 4 (N=8,362)
Age of smartphone ownership	n (%)	n (%)	n (%)
5	63 (0.9%)	49 (0.7%)	58 (0.8%)
6	71 (1.1%)	64 (0.9%)	68 (0.9%)
7	136 (2.2%)	117 (1.6%)	100 (1.3%)
8	346 (5.3%)	309 (4.3%)	276 (3.6%)
9	705 (10.6%)	535 (7.3%)	416 (5.2%)
10	1,936 (29.5%)	1,644 (21.9%)	1,312 (16.1%)
11	2,287 (32.6%)	2,337 (30.2%)	1,812 (21.3%)
12	1,104 (16.6%)	2,027 (25.3%)	2,447 (28.7%)
13	87 (1.2%)	569 (7.7%)	1,439 (16.7%)
14	--	23 (0.2%)	395 (5.0%)
15	2 (0.02%)	--	36 (0.5%)
16	--	1 (0.01%)	--

Sampling weights based on the American Community Survey were used to represent population estimates. Counts are unweighted and percentages are weighted, so raw counts may not match percentages.

© 2026 Nagata JM et al. *JAMA Network Open*.

eTable 5. Cross-sectional Associations Between Smartphone Ownership at Age 12 and Eating Disorder Symptoms at Age 12 in the Adolescent Brain Cognitive Development (ABCD) Study[a]

Smartphone ownership timing (exposure)	Binge-eating symptoms (N=4,988)		Inappropriate compensatory behaviors (N=4,988)		Worry about weight gain (N=4,988)		Self-worth tied to weight (N=4,988)		Any ED symptom (N=5,003)	
	PR (95% CI)	p	PR (95% CI)	p	PR (95% CI)	p	PR (95% CI)	p	PR (95% CI)	p
Age 12	**1.53 (1.19, 1.96)**	**0.001**	**1.54 (1.17, 2.03)**	**0.002**	1.65 (0.89, 3.08)	0.114	**2.37 (1.28, 4.37)**	**0.006**	**1.42 (1.19, 1.71)**	**<0.001**

[a]**Bold** indicates p<0.05. Sampling weights were applied to yield estimates based on the American Community Survey from the US Census. PR=prevalence ratio and CI=confidence interval. Models represent the abbreviated output from the generalized linear models without adjustment covariates.

© 2026 Nagata JM et al. *JAMA Network Open*.

eTable 6. Longitudinal Associations Between Smartphone Ownership at Age 12 and Eating Disorder Symptoms at Age 14 in the Adolescent Brain Cognitive Development (ABCD) Study[a]

Independent variables	Binge-eating symptoms (N=3,530)		Inappropriate compensatory behaviors (N=3,530)		Worry about weight gain (N=3,530)		Self-worth tied to weight (N=3,530)		Any ED symptom (N=3,756)	
	RR (95% CI)	p	RR (95% CI)	p	RR (95% CI)	p	RR (95% CI)	p	RR (95% CI)	p
Age 12	**1.42 (1.06, 1.90)**	**0.02**	**1.62 (1.33, 1.97)**	**<0.001**	1.41 (0.78, 2.56)	0.253	**2.21 (1.54, 3.18)**	**<0.001**	**1.56 (1.32, 1.84)**	**<0.001**

[a]**Bold** indicates p<0.05. Sampling weights were applied to yield estimates based on the American Community Survey from the US Census. RR=risk ratio and CI=confidence interval. Models represent the abbreviated output from the generalized linear models without adjustment covariates.

© 2026 Nagata JM et al. *JAMA Network Open*.

eTable 7. Cross-sectional Associations Between Smartphone Ownership at Age 12 and Eating Disorder Symptoms at Age 12 in the Adolescent Brain Cognitive Development (ABCD) Study[a]

	Binge-eating symptoms (N=3,612)		Inappropriate compensatory behaviors (N=3,612)		Worry about weight gain (N=3,612)		Self-worth tied to weight (N=3,612)		Any ED symptom (N=3,351)	
Independent variables	PR (95% CI)	p	PR (95% CI)	p	PR (95% CI)	p	PR (95% CI)	p	PR (95% CI)	p
Smartphone ownership	**1.60 (1.17, 2.19)**	**0.004**	1.23 (0.89, 1.70)	0.210	1.30 (0.59, 2.86)	0.520	2.03 (0.96, 4.30)	0.06	**1.32 (1.06, 1.64)**	**0.01**
Sex										
Female [reference]	--	--	--	--	--	--	--	--	--	--
Male	0.97 (0.74, 1.27)	0.824	1.11 (0.82, 1.50)	0.488	**0.42 (0.20, 0.88)**	**0.02**	**0.22 (0.11, 0.42)**	**<0.001**	0.95 (0.78, 1.16)	0.630
Race										
Black	0.84 (0.57, 1.24)	0.378	0.85 (0.57, 1.26)	0.422	0.99 (0.42, 2.30)	0.974	0.76 (0.36, 1.61)	0.467	0.92 (0.70, 1.20)	0.542
Other[a]	**0.64 (0.43, 0.94)**	**0.03**	0.77 (0.50, 1.18)	0.228	0.74 (0.32, 1.72)	0.482	0.81 (0.33, 1.96)	0.636	0.77 (0.58, 1.02)	0.07
White [reference]	--	--	--	--	--	--	--	--	--	--
Ethnicity										
Hispanic	1.08 (0.75, 1.56)	0.666	**1.53 (1.07, 2.19)**	**0.02**	1.96 (0.89, 4.29)	0.09	1.36 (0.63, 2.96)	0.436	1.24 (0.96, 1.60)	0.10
Non-Hispanic [reference]	--	--	--	--	--	--	--	--	--	--
Household income (Year 2)										
$49,999 or less	1.02 (0.70, 1.50)	0.905	1.07 (0.71, 1.61)	0.751	0.89 (0.35, 2.26)	0.808	1.45 (0.74, 2.85)	0.280	1.00 (0.76, 1.32)	0.985
$50,000 to $99,999	1.05 (0.77, 1.42)	0.772	1.32 (0.95, 1.84)	0.10	1.86 (0.95, 3.66)	0.07	1.71 (0.92, 3.20)	0.09	1.17 (0.95, 1.45)	0.147
$100,000 or greater [reference]	--	--	--	--	--	--	--	--	--	--
Parent/caregiver's highest education (Year 2)										
High school education or less	0.92 (0.58, 1.44)	0.710	1.07 (0.70, 1.64)	0.753	1.70 (0.80, 3.62)	0.171	0.59 (0.25, 1.38)	0.225	1.09 (0.81, 1.46)	0.585
College education or more [reference]	--	--	--	--	--	--	--	--	--	--
Child Behavior Checklist (Year 2)										
Attention-deficit/hyperactivity symptoms (t-score)	1.01 (0.98, 1.03)	0.632	0.99 (0.96, 1.02)	0.689	1.03 (0.98, 1.09)	0.209	0.98 (0.92, 1.04)	0.538	1.01 (0.99, 1.02)	0.456
Depressive symptoms (t-score)	**1.03 (1.01, 1.05)**	**0.003**	1.01 (0.98, 1.03)	0.567	**1.06 (1.02, 1.10)**	**0.004**	**1.06 (1.02, 1.10)**	**0.004**	**1.02 (1.00, 1.04)**	**0.01**
Device ownership (Year 2)										
Tablet	1.00 (0.78, 1.28)	0.996	1.07 (0.82, 1.40)	0.609	0.73 (0.42, 1.29)	0.280	1.11 (0.65, 1.90)	0.706	1.00 (0.84, 1.19)	0.999
Laptop	1.10 (0.86, 1.41)	0.450	0.84 (0.64, 1.09)	0.192	**1.79 (1.02, 3.14)**	**0.04**	0.84 (0.50, 1.41)	0.514	1.02 (0.86, 1.22)	0.811
iPod	1.08 (0.71, 1.65)	0.713	1.06 (0.68, 1.65)	0.785	2.05 (0.78, 5.36)	0.144	1.17 (0.47, 2.92)	0.739	1.11 (0.83, 1.49)	0.491
Smartwatch	**0.39 (0.18, 0.85)**	**0.02**	**1.79 (1.15, 2.77)**	**0.01**	2.08 (0.82, 5.27)	0.123	1.27 (0.53, 3.03)	0.593	1.15 (0.81, 1.63)	0.429
Pubertal status (Year 2)										
Pre/early-pubertal	**0.67 (0.48, 0.92)**	**0.02**	0.91 (0.65, 1.29)	0.611	1.67 (0.74, 3.77)	0.217	1.51 (0.71, 3.21)	0.281	0.87 (0.69, 1.10)	0.239
Mid/late/post-pubertal [reference]	--	--	--	--	--	--	--	--	--	--
Body weight (pounds)	**1.00 (1.00, 1.01)**	**0.02**	**1.01 (1.01, 1.01)**	**<0.001**	**1.01 (1.01, 1.02)**	**<0.001**	**1.01 (1.01, 1.02)**	**<0.001**	**1.01 (1.01, 1.01)**	**<0.001**
Parental monitoring (Year 2)	**0.66 (0.52, 0.83)**	**0.001**	0.84 (0.65, 1.08)	0.179	0.73 (0.42, 1.27)	0.270	**0.52 (0.36, 0.76)**	**0.001**	**0.76 (0.64, 0.90)**	**0.002**

© 2026 Nagata JM et al. *JAMA Network Open*.

[a]**Bold** indicates p<0.05. Sampling weights were applied to yield estimates based on the American Community Survey from the US Census. PR=prevalence ratio. CI=confidence interval. Models represent the abbreviated output from the generalized linear models with adjustment for sex, race, ethnicity, Year 2 household income, highest parental education, attention-deficit/hyperactivity symptoms, depressive symptoms, tablet ownership, laptop ownership, iPod ownership, smartwatch ownership, parental monitoring, pubertal status, body weight, and study site.

© 2026 Nagata JM et al. *JAMA Network Open*.

eTable 8. Longitudinal Associations Between Smartphone Ownership at Age 12 and Eating Disorder Symptoms at Age 14 in the Adolescent Brain Cognitive Development (ABCD) Study[a]

	Binge-eating symptoms (N=2,128)		Inappropriate compensatory behaviors (N=2,128)		Worry about weight gain (N=2,128)		Self-worth tied to weight (N=2,128)		Any ED symptom (N=2,221)	
Independent variables	RR (95% CI)	p	RR (95% CI)	p	RR (95% CI)	p	RR (95% CI)	p	RR (95% CI)	p
Smartphone ownership	**1.57 (1.05, 2.33)**	**0.03**	**1.50 (1.17, 1.91)**	**0.001**	1.10 (0.56, 2.17)	0.784	**2.00 (1.31, 3.06)**	**0.001**	**1.52 (1.23, 1.88)**	**<0.001**
Sex										
Female [reference]	--	--	--	--	--	--	--	--	--	--
Male	**0.53 (0.36, 0.76)**	**0.001**	**0.60 (0.47, 0.77)**	**<0.001**	**0.28 (0.12, 0.67)**	**0.004**	**0.28 (0.17, 0.45)**	**<0.001**	**0.62 (0.50, 0.75)**	**<0.001**
Race										
Black	0.73 (0.40, 1.34)	0.308	1.09 (0.79, 1.49)	0.608	1.23 (0.45, 3.37)	0.688	1.14 (0.68, 1.92)	0.612	0.98 (0.73, 1.31)	0.892
Other[a]	1.04 (0.67, 1.62)	0.851	1.30 (0.99, 1.70)	0.06	**3.01 (1.31, 6.95)**	**0.01**	1.45 (0.93, 2.28)	0.104	1.14 (0.90, 1.45)	0.274
White [reference]	--	--	--	--	--	--	--	--	--	--
Ethnicity										
Hispanic	1.12 (0.72, 1.74)	0.620	1.29 (0.97, 1.70)	0.08	1.03 (0.43, 2.44)	0.955	1.58 (0.94, 2.66)	0.09	1.27 (0.99, 1.64)	0.06
Non-Hispanic [reference]	--	--	--	--	--	--	--	--	--	--
Household income (Year 2)										
$49,999 or less	1.33 (0.84, 2.12)	0.226	0.88 (0.65, 1.19)	0.402	0.78 (0.29, 2.10)	0.623	0.88 (0.53, 1.45)	0.612	1.04 (0.81, 1.35)	0.750
$50,000 to $99,999	0.83 (0.55, 1.25)	0.365	0.86 (0.66, 1.10)	0.225	0.46 (0.20, 1.04)	0.06	0.75 (0.48, 1.16)	0.191	0.87 (0.70, 1.09)	0.223
$100,000 or greater [reference]	--	--	--	--	--	--	--	--	--	--
Parent/caregiver's highest education (Year 2)										
High school education or less	1.07 (0.66, 1.74)	0.782	0.87 (0.63, 1.20)	0.398	1.51 (0.54, 4.25)	0.432	1.26 (0.77, 2.05)	0.361	0.90 (0.67, 1.19)	0.453
College education or more [reference]	--	--	--	--	--	--	--	--	--	--
Child Behavior Checklist (Year 2)										
Attention-deficit/hyperactivity symptoms (t-score)	1.00 (0.97, 1.03)	0.882	1.01 (0.99, 1.03)	0.396	1.02 (0.96, 1.08)	0.461	1.02 (0.99, 1.05)	0.258	1.00 (0.98, 1.02)	0.825
Depressive symptoms (t-score)	1.03 (1.00, 1.05)	0.05	1.01 (0.99, 1.03)	0.253	1.04 (0.98, 1.09)	0.187	1.01 (0.98, 1.04)	0.622	**1.02 (1.00, 1.03)**	**0.04**
Device ownership (Year 2)										
Tablet	1.20 (0.88, 1.63)	0.249	0.94 (0.77, 1.15)	0.548	1.79 (0.86, 3.73)	0.121	1.18 (0.85, 1.65)	0.331	1.03 (0.87, 1.22)	0.756
Laptop	**0.57 (0.40, 0.81)**	**0.002**	0.91 (0.74, 1.12)	0.389	**0.42 (0.20, 0.85)**	**0.02**	0.85 (0.60, 1.20)	0.347	**0.83 (0.69, 0.99)**	**0.04**
iPod	0.94 (0.51, 1.72)	0.840	1.25 (0.90, 1.73)	0.187	0.68 (0.15, 3.07)	0.619	1.45 (0.87, 2.43)	0.157	1.19 (0.89, 1.58)	0.234
Smartwatch	1.12 (0.58, 2.16)	0.728	1.16 (0.80, 1.69)	0.438	1.32 (0.48, 3.60)	0.594	1.04 (0.52, 2.08)	0.905	1.11 (0.80, 1.55)	0.530
Pubertal status (Year 2)										
Pre/early-pubertal	1.08 (0.71, 1.63)	0.726	0.86 (0.65, 1.14)	0.299	1.37 (0.50, 3.76)	0.545	1.01 (0.61, 1.68)	0.977	0.95 (0.75, 1.20)	0.664
Mid/late/post-pubertal [reference]	--	--	--	--	--	--	--	--	--	--
Body weight (pounds)	**1.01 (1.00, 1.01)**	**0.005**	**1.01 (1.00, 1.01)**	**<0.001**	**1.01 (1.00, 1.01)**	**0.02**	**1.01 (1.00, 1.01)**	**<0.001**	**1.01 (1.00, 1.01)**	**<0.001**
Parental monitoring (Year 2)	0.94 (0.68, 1.30)	0.704	0.94 (0.75, 1.19)	0.610	0.81 (0.47, 1.39)	0.444	1.27 (0.87, 1.87)	0.218	0.98 (0.81, 1.18)	0.800
Eating disorder (ED) symptoms (Age 12)										
Binge-eating symptoms	**2.64 (1.83, 3.82)**	**<0.001**	--	--	--	--	--	--	--	--
Compensatory behaviors	--	--	**1.82 (1.39, 2.37)**	**<0.001**	--	--	--	--	--	--

© 2026 Nagata JM et al. *JAMA Network Open*.

Worry about weight gain	--	--	--	--	**4.65 (1.47, 14.69)**	**0.009**	--	--	--	--
Self-worth tied to weight	--	--	--	--	--	--	**4.75 (3.06, 7.38)**	**<0.001**	--	--
Any ED symptom	--	--	--	--	--	--	--	--	**1.65 (1.38, 1.99)**	**<0.001**

[a]**Bold** indicates p<0.05. Sampling weights were applied to yield estimates based on the American Community Survey from the US Census. RR=risk ratio. CI=confidence interval. Models represent the abbreviated output from the generalized linear models with adjustment for sex, race, ethnicity, Year 2 household income, highest parental education, attention-deficit/hyperactivity symptoms, depressive symptoms, tablet ownership, laptop ownership, iPod ownership, smartwatch ownership, parental monitoring, pubertal status, body weight, eating disorders at age 12, and study site.

© 2026 Nagata JM et al. *JAMA Network Open*.

eReferences

1. Garavan H, Bartsch H, Conway K, et al. Recruiting the ABCD sample: design considerations and procedures. *Dev Cogn Neurosci*. 2018;32:16-22. doi:10.1016/j.dcn.2018.04.004

2. ABCD Consortium. 6.0 data release. 2025. Accessed July 15, 2026. https://docs.abcdstudy.org/latest/documentation/release_notes/6_0.html

3. Carvalho C, Koss K, Ravindran N. The role of smartphones in adolescent-parent discrepancy in reporting adolescents' internalizing problems. *Dev Psychopathol*. Published online September 8, 2025:1-13. doi:10.1017/S0954579425100618

4. Richter A, Adkins V, Selkie E. Youth perspectives on the recommended age of mobile phone adoption: survey study. *JMIR Pediatr Parent*. 2022;5(4):e40704. doi:10.2196/40704

5. Townsend L, Kobak K, Kearney C, et al. Development of three web-based computerized versions of the Kiddie Schedule for Affective Disorders and Schizophrenia child psychiatric diagnostic interview: preliminary validity data. *J Am Acad Child Adolesc Psychiatry*. 2020;59(2):309-325. doi:10.1016/j.jaac.2019.05.009

6. Makowski C, Westwater ML, Rhee KE, Zou J, Bischoff-Grethe A, Wierenga CE. Sociodemographic correlates of parent and youth-reported eating disorder symptoms in the Adolescent Brain Cognitive Development Study. *medRxiv*. Published online June 15, 2024:2023.12.18.23300155. doi:10.1101/2023.12.18.23300155

7. Barzilay R, Pimentel SD, Tran KT, Visoki E, Pagliaccio D, Auerbach RP. Smartphone ownership, age of smartphone acquisition, and health outcomes in early adolescence. *Pediatrics*. Published online December 1, 2025:e2025072941. doi:10.1542/peds.2025-072941

8. Al-Shoaibi AAA, Shao IY, Ganson KT, et al. Prospective association of screen time with binge-eating disorder among adolescents in the United States: the mediating role of depression. *Int J Eat Disord*. 2024;57(5):1192-1201. doi:10.1002/eat.24169

9. Bläschke L, Weisner FE, Hinney A, et al. Screen Time and Puberty Timing: Findings from the Adolescent Brain Cognitive Development Study. *medRxiv*. Published online January 28, 2026:2026.01.26.26344850. doi:10.64898/2026.01.26.26344850

10. Murray SB, Blashill AJ, Calzo JP. Prevalence of Disordered Eating and Associations With Sex, Pubertal Maturation, and Weight in Children in the US. *JAMA Pediatr*. 2022;176(10):1039-1040. doi:10.1001/jamapediatrics.2022.2490

11. Al-Shoaibi AAA, Lavender JM, Kim SJ, et al. Association of body mass index with progression from binge-eating behavior into binge-eating disorder among adolescents in the United States: a prospective analysis of pooled data. *Appetite*. 2024;200:107419. doi:10.1016/j.appet.2024.107419

© 2026 Nagata JM et al. *JAMA Network Open*.

12. Martinson LE, Esposito-Smythers C, Blalock DV. The Effect of Parental Monitoring on Trajectories of Disordered Eating Attitudes and Behaviors Among Adolescents: An Individual Growth Curve Analysis. *Appetite*. 2016;107:180-187. doi:10.1016/j.appet.2016.07.035

13. Hu Y ting, Wang Q. Self-control, parental monitoring, and adolescent problematic mobile phone use: testing the interactive effect and its gender differences. *Front Psychol*. 2022;13:846618. doi:10.3389/fpsyg.2022.846618

© 2026 Nagata JM et al. *JAMA Network Open*.
