# Supplementary material for: Smartphone Ownership and Eating Disorder Symptoms in Early Adolescence
Source: JAMA Netw Open. 2026 Sep 25;9(9):e2637897. doi: 10.1001/jamanetworkopen.2026.37897 (PMC13615465; doi:10.1001/jamanetworkopen.2026.37897)
Supplement: Supplementary file 2 [file jamanetwopen-e2637897-s002.pdf]

Data Sharing Statement

Nagata. Smartphone Ownership and Eating Disorder Symptoms in Early Adolescence. *JAMA Netw Open*. Published September 25, 2026. doi:10.1001/jamanetworkopen.2026.37897

Data
Data available: Yes
Data types: Deidentified participant data
How to access data: Data used in the preparation of this article were obtained from the ABCD Study (https://abcdstudy.org), held in the NIH Brain Development Cohorts (NBDC) Portal (https://nbdc.nida.nih.gov/)
When available: With publication

Supporting Documents
Document types: None

Additional Information
Who can access the data: Researchers whose proposed use of the data has been approved
Types of analyses: For a specified purpose
Mechanisms of data availability: With a signed data access agreement
